# High School Students as Citizen Scientists to Decrease Radon Exposure

**DOI:** 10.3390/ijerph17249178

**Published:** 2020-12-08

**Authors:** Ellen J. Hahn, Craig Wilmhoff, Mary Kay Rayens, Nicholas B. Conley, Emily Morris, Angela Larck, Trista Allen, Susan M. Pinney

**Affiliations:** 1 UK-CARES and BREATHE, College of Nursing, University of Kentucky, Lexington, KY 40504 USA; 2 Perry County Central High School, Hazard, KY 41701, USA; craig.wilmhoff@perry.kyschools.us; 3 BREATHE, College of Nursing, University of Kentucky, Lexington, KY 40504 USA; mkrayens@uky.edu (M.K.R.); nicholas.conley@uky.edu (N.B.C.); 4 Kentucky Geological Survey, University of Kentucky, Lexington, KY 40505, USA; EmilyMorris@uky.edu; 5 Center for Environmental Genetics, University of Cincinnati, Cincinnati, OH 45267, USA; larckan@ucmail.uc.edu (A.L.); susan.pinney@uc.edu (S.M.P.); 6 Fairfield Senior High School, Fairfield, OH 45014, USA; tristaallen05@gmail.com

**Keywords:** radon, citizen science, lung cancer, cancer prevention, youth-engaged

## Abstract

Residents in rural Kentucky (KY) and suburban Ohio (OH) expressed concerns about radon exposure and lung cancer. Although 85% of lung cancer cases are caused by tobacco smoke, radon exposure accounts for 10–15% of lung cancer cases. Academic and community members from the University of KY and the University of Cincinnati developed and pilot-tested a family-centered, youth-engaged home radon testing toolkit. The radon toolkit included radon information, and how to test, interpret, and report back findings. We educated youth as citizen scientists and their teachers in human subjects protection and home radon testing using the toolkit in the classroom. Youth citizen scientists explained the study to their parents and obtained informed consent. One hundred students were trained in human subjects protection, 27 had parental permission to be citizen scientists, and 18 homeowners completed surveys. Radon values ranged from < 14.8 Bq/m^3^ to 277.5 Bq/m^3^. Youth were interested and engaged in citizen science and this family-centered, school-based project provided a unique opportunity to further the healthy housing and quality education components of the Sustainable Development Goals for 2030. Further research is needed to test the impact of student-led, family-centered citizen science projects in environmental health as part of school curricula.

## 1. Introduction

Radon is a naturally occurring radioactive gas that is odorless, tasteless, and colorless. Radon emanates from the soil and is influenced by geological formations. The BREATHE (Bridging Research Efforts and Advocacy Toward Healthy Environments) team at the University of Kentucky (UK) reported significant correlations between bedrock type and indoor radon test results in KY [1,2]. Radon exposure accounts for 10–15% of lung cancer cases and it causes an estimated 21,000 lung cancer deaths in the U.S. annually [3]. Testing for radon is a low-cost and simple procedure. Radon testing is the only way to measure indoor levels and exposure risks. However, radon testing rates remain low in the U.S. [1,2].

Citizen science may be one approach to increase radon testing rates. Citizen science is a process in which community members are trained as researchers to conduct community-based monitoring, data collection, and community-based planning and management of resources [4]. Citizen science projects enable and empower community members to actively participate in multidisciplinary research. Citizen scientists access their own data and the collective data generated by others in order to report back the findings and plan effective solutions to community problems [4]. When citizen scientists contribute to community-academic partnerships, they are effective in enriching research designs collecting sound data, and reporting back research findings and their health implications [5,6,7].

Community members in rural southeastern Kentucky (KY) and suburban southwest Ohio (OH) expressed concern about radon exposure given high rates of lung cancer, high prevalence of smoking and exposure to secondhand smoke (SHS), increased use of fracking in the region, and bedrock geology known to produce radon. Based on our previous experiences conducting collaborative community-engaged research with high school students for environmental risk reduction, the Community Engagement Cores (CEC) at the UK Center for Appalachian Research in Environmental Sciences (UK-CARES) and the Center for Environmental Genetics at UC recruited high school science teachers for this project. These teachers (one in a rural Appalachian community and one in a suburban area in southwest OH) worked with us to address radon exposure using a citizen science approach with youth. Using this type of approach with adults increases the prevalence of radon testing. Casey et al. identified community champions, or citizen scientists, to provide radon information to community members, support testing, be key points of contact, and report back findings to community members [8]. The project resulted in a 97% response rate for radon testing. Radon testing rates increased when engaging citizen scientists in the research. In another citizen science study, Ablah et al. recruited nine community leaders with an interest in the environment to be part of the project design team to provide direction on project implementation [9]. Citizen scientists became members of the environmental leadership council, and they assisted in categorizing and prioritizing environmental concerns identified during 52 discussion groups. The citizen scientists identified radon as an environmental concern [9]. 

We were not able to find any studies that engaged youth as citizen scientists to increase radon testing. However, collaborating with youth citizen scientists presents opportunities and potential benefits for both the youth and the research institution [10,11,12]. Engaging youth in research promotes youth empowerment and enhances the relationship between communities and academic institutions [11,13]. The purpose of this exploratory project was to assess the feasibility of the citizen science approach by engaging youth in the school setting to raise awareness of home radon testing in rural KY and suburban OH. A secondary aim was to assess the agreement between indoor radon values and measurements taken exterior to the home using soil samples in KY.

## 2. Materials and Methods

This prospective descriptive feasibility project used a citizen science approach to promote radon testing [4]. First, we educated students enrolled in college-preparatory biology classes in one high school located in rural Appalachian KY and one high school in suburban OH in human subjects protection principles and the basics of radon exposure. We then invited the students to join our project team. For those who were interested, we provided documents (e.g., parent consent form, individual investigator agreement) for them to take home for parent signature. For those who returned the signed documents, we provided more in-depth education about the health effects of radon, radon testing and mitigation, and the process of recruiting and consenting a parent or other significant adult to test the home for radon. We offered the youth citizen scientists in KY the option of soil sampling for radon outside the home by geologists from the KY Geological Survey. Due to limited funding, we were unable to offer soil sampling in OH. Following radon testing, the youth citizen scientists reported back the results to the homeowners, with help of the academic team, and discussed mitigation options for those with high radon. 

Student classroom education. We invited students to attend a total of three classroom-based education sessions: (1) Human Subjects Protection; (2) Radon Overview and Radon Toolkit (see Supplementary Materials) Testing Protocol, and; (3) Evaluating Radon Data and Discussing Results. In advance of the first session, we assigned students to read an overview of the history of human subjects protection. Next, they completed the IRB-approved CIRT [14] classroom session. At the end of the session, we distributed to each student individual investigator agreements forms, minor consent forms, and parent consent forms and invited them to obtain their parent’s signature to participate. Once the students returned all three forms, we added them to the IRB-approved study protocol as key personnel. Students were each asked to recruit one homeowner, either their parent or another adult, to complete a brief survey and test their home for radon. Adult participation in the study required home-ownership.

In the second (2) classroom session, we presented radon facts and information on the health effects of radon. We reviewed step-by-step instructions on deploying the short-term charcoal-based radon test kit and best practices when communicating with study participants. Following session #2, we provided to the students who had returned all parent-signed permission forms a Radon Toolkit including: radon test kit booklet; radon and tobacco smoke synergistic risk infographic fact sheet; county-specific geologic radon potential map [15]; letter to residents explaining soil testing (rural Appalachian KY school only), and; the Alpha Energy charcoal-based passive short-term detector [16]. 

The third (3) classroom session occurred after home radon testing was completed. We shared each school group’s data as a whole and provided each youth citizen scientist their homeowner’s individual data. We helped them interpret the radon values, and shared the skills needed to effectively communicate the radon values and discuss the next steps with the study participants.

Homeowner indoor radon testing. After explaining the study and consenting the homeowner, youth citizen scientists invited the homeowner to complete a brief 15-item online survey to assess home characteristics and perceived risk related to radon (see below). Homeowner participants, with the help of the youth citizen scientists, deployed the Alpha Energy charcoal-based, short-term passive radon detector for at least 2 days and no more than 4 days. Homeowners then returned the test kits via prepaid postal mail to the laboratory for analysis. Homeowners in KY and OH tested in February-April during the wet, cold months. For homeowners in rural Appalachian KY who opted-in to the soil gas sampling, we contacted the homeowner to discuss a sampling time window, access to the property, and safe sampling environment (e.g., securing pets during the testing window). 

**Figure 1:**
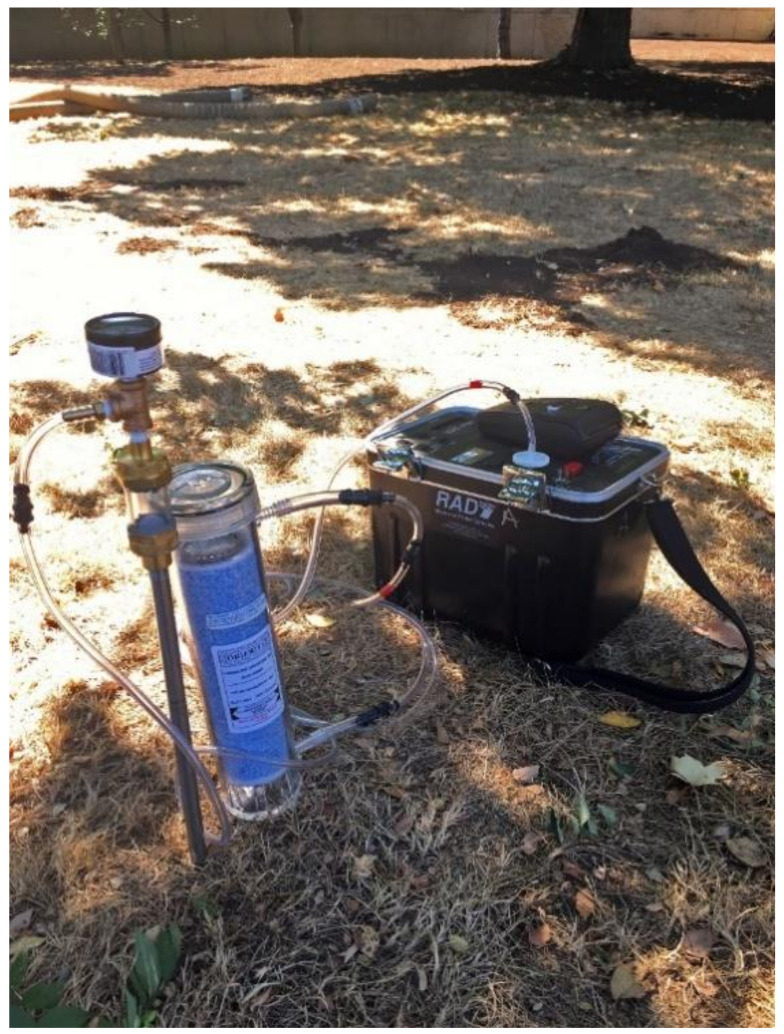
RAD7 soil gas testing setup.

Soil radon measurements. The geologists collected soil gas radon concentration samples outside the homes of participating homeowners in rural Appalachian KY. Although we attempted to align the time of soil sampling with the end of the indoor testing period, we conducted outdoor tests between 1 and 13 days after homeowners completed the corresponding indoor test. The goal for outdoor testing was to take samples 3.05–4.57 m away from the home at a depth of 0.61 m, though this was not always possible due to rocky or impacted soil. We collected one soil gas sample for each participating home using the DURRIDGE RAD7 electronic radon detector. The RAD7 measured the average radon concentration in Bq/m or pCi/L. We used CAPTURE software to correct for high relative humidity levels for any measurements where the humidity inside the RAD7 was higher than ten percent. The RAD7 was connected to a soil probe and pulled samples of air into a chamber for analysis (Figure 1).

Surveys of homeowners. We invited homeowners in both locations to complete a brief online survey immediately prior to deploying their radon test kit using a personal computer, smart phone, or tablet. The survey items assessed home characteristics (years they had lived there, year built, housing, and foundation type) as well as family characteristics (e.g., the number living in the home, the number who currently smoke). Survey items assessed the perceived seriousness of illnesses caused by radon, the likelihood of radon being in the place they live, and the perceived risk of lung cancer in their lifetime. The survey also assessed self-efficacy related to mitigation including whether they would be able to fix their home in the event of high radon levels; whether they would have the money to do so; and how easy it would be to reduce high radon levels. Finally, we asked homeowner participants in KY if a geologist could take soil samples from their property; given funding limitations, we did not offer soil sampling to the OH homeowners. 

Report back. The laboratory returned the indoor air results and the geologists returned the soil radon results to the University of Kentucky academic team; based on these data, we assembled and distributed personalized radon report back folders to each of the youth citizen scientists. The folders included a graph displaying the indoor radon test results by school group (see Supplementary Materials). We sent the group results for their school as well as their individual homeowner’s radon value (circled) to each citizen scientist. This allowed students to compare the group’s radon values without the risk of identifying individual study participants. For the rural Appalachian KY students and homeowners, we provided a companion graph displaying soil radon gas values. We also included an information sheet in the radon report back folder that interpreted the result, next steps, and contact information for UK’s BREATHE Radon Policy Research Program. 

For homeowners who tested at or above the U.S. Environmental Protection Agency’s EPA action level of 4.0 pCi/L, or 148 Bq/m^3^, we offered a $1000 voucher toward a radon mitigation system. If they were interested in talking with us about radon mitigation, we would offer help in identifying a certified radon measurement and mitigation company that could provide an estimate. For homeowners who tested at or above the World Health Organization’s (WHO) action level of 100 Bq/m^3^, but less than the U.S. EPA action level, we sent them a long-term charcoal-based test kit (90 days to one year). 

Process evaluation of the citizen science experience. We invited all OH students who had participated in at least one education session to complete a process evaluation survey post-project; we were not able to invite the KY students to participate in this evaluation as it was too late in the semester and we were unable to reach the students. The evaluation survey items assessed which classroom sessions they attended and their satisfaction with each. They rated the degree to which the individual classroom sessions improved knowledge and provided information, and to what extent the presenter’s delivery was clear. One survey item asked whether or not they had consented a homeowner to participate and, if not, what barriers existed. Students could add comments in response to an open-ended item.

Data Analysis. Our primary analysis strategy for the descriptive study was univariate statistics, including means and standard deviations or frequency distributions. In addition, we summarized the degree of agreement between indoor and outdoor soil radon assessments using graphical methods. For homes with both indoor and outdoor measurements, we used Spearman’s rank correlation to evaluate the degree of association between them. We used SAS, v. 9.4 for all analyses. 

## 3. Results

Of the 100 students whom we educated in human subjects protection, 27% returned signed forms from their parents to participate. The participation rate was the same in rural and suburban schools. Of the 27 youth citizen scientists who participated, a total of 18 homeowners completed surveys (67%), including 13 in KY and five in OH. On average, they had lived in their homes for 13.2 years (*SD* = 5.7), and the average age of the home was 28.4 years (*SD* = 16.7). The majority (78%) lived in a single family home unattached to any other dwelling; the remainder lived in a mobile home (22%). Most participants (78%) indicated four people lived in their current residence; other responses were two (6%), three (11%), or five (6%) people living in the home. Nearly all (89%) indicated there was no one in the household who currently smoked cigarettes, cigars, or pipes.

As shown in Table 1, more than half (56%) of the homeowners indicated that illnesses caused by radon were ‘very serious’ or ‘extremely serious’; another 22% viewed radon as ‘serious’. However, over three-fourths (78%) of homeowners thought it was either ‘very unlikely’ or ‘unlikely’ that radon would be present in their home. On a scale of 0 to 10, with 0 being lowest risk and 10 highest risk, over half of homeowners (56%) rated their perceived risk of developing lung cancer in their lifetime as a 0 or 1. None of the homeowners indicated a score of 7–10 in response to this item. With regard to mitigation, nearly two-thirds (61%) were neutral on whether they thought they would be able to fix their home for high radon levels to prevent lung cancer. Regarding the financial implications of mitigation, nearly all (89%) disagreed or were neutral in response to whether they had the money to fix their home for high radon. Concerning whether a fix for radon would be easy for the homeowner, nearly all were neutral (89%).

Of the 18 homeowners who completed surveys, 15 had valid short-term radon test results ranging from < 14.8 Bq/m^3^ to 277.5 Bq/mq^3^. Two of the 15 tests were at or above the EPA action level; three tests exceeded the WHO action level of 100 Bq/m^3^. While we offered mitigation vouchers to the homeowners who tested at or above the EPA action level of 148 Bq/m^3^, neither were interested in having their homes mitigated.

The geologists measured radon gas concentrations in soil on each participating property (Appalachian KY group only) over a 30-min testing period. We had measurements for both indoor and soil radon from a total of eight homes. The limited number of homes with both measurements was due to three KY homeowners opting out of soil testing and no soil testing in any of the OH homes (due to limited funding). There was variability in air radon levels inside the home and in the soil outside the home (see Table 2). For the eight homes with complete testing data, the average indoor radon measurement was 79.1 Bq/m^3^ (ranging from 14.8 to 277.5 Bq/m^3^), while the average soil measurement was 18,916 Bq/m^3^ (ranging from 1036 to 76,405 Bq/m^3^). The degree of association between these two measurements was not significant (rho = −0.13, *p* = 0.76). 

Eight of the 30 students (27%) who participated in the OH educational sessions completed an evaluation survey for at least one of the three sessions. Of those who evaluated the ‘Human Subjects Protection’ session, nearly two-thirds (63%) were satisfied (either ‘extremely’ or ‘somewhat’) with the session, and 63% were ‘extremely’ or ‘somewhat’ satisfied with the overall learning experience (see Table 3). Students rated the other two classroom sessions, ‘Radon Overview and Radon Toolkit Testing Protocol’ and ‘Evaluating Radon Data and Discussing Results’ similarly. For each of these two sessions, citizen scientists were ‘extremely’ or ‘somewhat’ satisfied with the overall classroom experience and nearly all (80%) were satisfied with the learning experience. In terms of the increase in knowledge or skills, the provision of useful information, and the evaluation of the session presenter, students provided positive assessments for each of these items for all three classroom sessions at least 86% of the time. In particular, the majority of students either ‘strongly’ or ‘somewhat’ agreed with each of the positive statements of each session. Participant refusal was the primary reason for not being able to consent a homeowner. Of the eight OH students who had permission to be citizen scientists, 75% indicated they had consented a homeowner to participate in the study. The positive open-ended feedback from the students included: “I found the learning/training sessions to be highly informational and I feel prepared to partake in more citizen science studies,” and “I loved this experience. How can I get more involved in the future?” Barriers included: “I wanted to participate but was not allowed by my Dad,” and “The classroom sessions provided useful information but it was usually boring”.

## 4. Discussion

Although we engaged more students in the rural Appalachian KY school in the initial classroom session, just over one-fourth (27%) in both rural and suburban locations returned the parent-signed forms and became youth citizen scientists. Among the youth citizen scientists, we documented high rates of returned test kits and valid results. Our results using a citizen science approach, similar to other youth-focused community-engaged projects, demonstrated that youth participation can improve outcomes (in this case, radon testing) [10,11,12,13,17]. This project was consistent with the healthy environment dimension of the United Nations’ Sustainable Development Goals (SDG) for 2030. Specifically, SDG Goal 11 is to make communities inclusive, safe, resilient, and sustainable (11.1 access to adequate, safe, and affordable housing). Further, SDG Goal 3 (ensuring healthy lives and promoting well-being) and Goal 4 (inclusive and equitable quality education) provided context for the significance of the radon awareness project described here. We found the citizen science approach with youth to promote healthy housing (i.e., raising radon awareness) as a feasible education method in both rural and urban communities [18].

Homeowners in this study did not view themselves as susceptible to radon exposure; nearly 8 of 10 homeowners did not think it likely that radon would be present in their home. Perceived risk of developing lung cancer was also low, with over half indicating low risk. Six of 10 homeowners were neutral or unsure, in their ability to mitigate their home for radon. One contributing factor to the lack of self-efficacy for radon mitigation could be the financial burden; nearly all respondents reported not having the money to mitigate their home. Testing for radon is essential but property owners with high levels of radon need access to affordable radon mitigation. Although we offered financial incentives to mitigate for those with high radon, research is needed to understand the decision to remediate or not remediate environmental hazards [19].

Several strengths contributed to the success of the study. First, we collaborated with teachers who had community-academic research experience and that was very helpful throughout the project. The teacher partners were also active members of their communities and had solidified relationships within their schools. Second, this was a cross-institution project between two NIEHS-funded Environmental Health Science Core Centers: the University of Kentucky and the University of Cincinnati. Having a strong relationship between these two flagship universities allowed access to more resources than would normally be available at one institution. This cross-institution collaboration and longstanding trust in each of the rural and suburban communities led to the project’s success. Third, youth citizen scientists were actively engaged and positive throughout the project. Their participation in the classroom sessions, interest in the project and the research process, and participation in professional and community presentations [20,21], were all factors that contributed to the success of this project. 

There were also limitations to the findings. First, the suburban OH school was located in a racially and ethnically diverse community with a high proportion of non-English speaking students and renters. A higher proportion of the OH students lived in rental property, compared to the sample of rural Appalachian KY students. Since our protocol required the adult to own their home in order to be eligible for the project, the disparity in home-ownership may have contributed to the overall participation rates. Second, the indoor radon testing and soil sample measurements did not occur concurrently. During the time between indoor radon sampling and soil sampling, a number of factors could have affected soil radon levels including rainfall, barometric pressure, and temperature [22,23,24,25,26]. Third, we were unable to talk directly with the homeowners who had high radon to assess factors related to their disinterest in using the mitigation voucher as the focus of the project was on educating youth as citizen scientists vs. a community campaign to reduce radon risk. Lastly, we encountered several challenges. Due to the coronavirus pandemic, the in-person educational sessions were rescheduled as virtual meetings. Also, some community members in the OH site expressed social stigma toward radon exposure/lung cancer and this may have impacted study participation and willingness to test for radon in the home. Future research is needed to communicate that radon is a naturally occurring radioactive gas and not a result of individual behaviors and that testing is the only way to determine radon levels.

## 5. Conclusions

Engaging youth in a citizen science project to increase radon home testing was feasible and effective. In both instances (rural KY and suburban OH), nearly three in 10 students became citizen scientists. Of the students who participated, over half returned a radon test kit that yielded valid results. The association between indoor air and soil gas measurements for radon was not significant, but this needs to be evaluated further with a larger sample and with attention to the timing of measurement and weather indices that may influence soil gas radon values.

## 6. Future Intentions

This exploratory feasibility project set the stage for future research testing citizen science approaches with youth in schools to increase radon testing and mitigation. While we offered a mitigation voucher to the homeowners who tested at or above the EPA action level of 4.0 pCi/L, they were not interested in having their homes mitigated. Future research is needed to explore barriers toward mitigation and ways to enhance self-efficacy toward radon mitigation. Research is also needed to test the effects of student-led, family-centered citizen science projects as part of school curricula. Youth interest and engagement in citizen science in projects like this one suggest that it would be useful to measure the short- and long-term impacts of citizen science in schools on individual, organization, and community components of youth engagement [25]. Engaging youth in citizen science can provide an opportunity to promote community-engaged research and outreach that may not be available by using more traditional research methods.

## Figures and Tables

**Table 1:** Homeowners’ perception of severity, risk, and ability to mitigate for radon (N = 18).

Perception Variable	n (%)
How serious are illnesses caused by radon?	
Not serious at all	3 (16.7)
Somewhat serious	1 (5.6)
Serious	4 (22.2)
Very serious	6 (33.3)
Extremely serious	4 (22.2)
What is the likelihood of there being radon in the place where you live?	
Very likely	6 (33.3)
Somewhat likely	8 (44.4)
Somewhat unlikely	3 (16.7)
Very unlikely	1 (5.6)
How would you rate your risk of developing lung cancer in your lifetime, on a scale of 0–10 where 0 is the lowest and 10 is the highest risk?	
0	7 (38.9)
1	3 (16.7)
2	1 (5.6)
3	3 (16.7)
4	2 (11.1)
5	1 (5.6)
6	1 (5.6)
7–10	0 (0.0)
I am able to fix my home for high radon to prevent lung cancer.	
Strongly disagree	1 (5.6)
Disagree	1 (5.6)
Neutral	11 (61.1)
Agree	4 (22.2)
Strongly agree	1 (5.6)
I have the money to fix my home for high radon to prevent lung cancer.	
Strongly disagree	6 (33.3)
Disagree	6 (33.3)
Neutral	4 (22.2)
Agree	1 (5.6)
Strongly agree	1 (5.6)
I can easily fix my home for high radon to prevent lung cancer.	
Strongly disagree	3 (16.7)
Disagree	6 (33.3)
Neutral	7 (38.9)
Agree	2 (11.1)
Strongly agree	0 (0.0)

**Table 2:** Indoor and soil radon measurements by home ID#.

Home ID#	Indoor Radon Level (Bq/m^3^)	Soil Radon Level (Bq/m^3^)
1	37.0	18,167
2	277.5	1036
3	122.1	15,059
4	22.2	1295
5	14.8	1443
6	22.2	32,597
7	96.2	5328
8	40.7	76,405
9	22.2	--
10	22.2	--
11	22.2	--
12	--	1480
13	192.4	--
14	107.3	--
15	133.2	--
16	44.4	--

**Table 3:** Ohio Student Evaluations of the Three Classroom Sessions (*n* = 8).

Rating Items	Classroom Education Sessions		
	Protecting Human Subjects n (%)	Radon Health Risks/Radon Testing n (%)	Interpreting/Discussing Radon Results n (%)
Overall, how would you rate the training session?			
Extremely satisfied	1 (12.5)	0 (0.0)	1 (20.0)
Somewhat satisfied	4 (50.0)	5 (100.0)	4 (80.0)
Somewhat dissatisfied	0 (0.0)	0 (0.0)	0 (0.0)
Extremely dissatisfied	3 (37.5)	0 (0.0)	0 (0.0)
Overall, how would you rate your learning experience?			
Extremely satisfied	1 (12.5)	1 (20.0)	0 (0.0)
Somewhat satisfied	4 (50.0)	3 (60.0)	5 (100.0)
Somewhat dissatisfied	1 (12.5)	0 (0.0)	0 (0.0)
Extremely dissatisfied	2 (25.0)	1 (20.0)	0 (0.0)
My knowledge or skills have improved through the training session			
Strongly agree	1 (14.3)	3 (60.0)	1 (20.0)
Somewhat agree	5 (71.4)	2 (40.0)	4 (80.0)
Somewhat disagree	1 (14.3)	0 (0.0)	0 (0.0)
Strongly disagree	0 (0.0)	0 (0.0)	0 (0.0)
The training session provided useful information			
Strongly agree	3 (37.5)	5 (100.0)	3 (60.0)
Somewhat agree	4 (50.0)	0 (0.0)	2 (40.0)
Somewhat disagree	1 (12.5)	0 (0.0)	0 (0.0)
Strongly disagree	0 (0.0)	0 (0.0)	0 (0.0)
The presenter clearly communicated the information			
Strongly agree	3 (42.9)	3 (60.0)	3 (60.0)
Somewhat agree	3 (42.9)	2 (40.0)	2 (40.0)
Somewhat disagree	1 (14.3)	0 (0.0)	0 (0.0)
Strongly disagree	0 (0.0)	0 (0.0)	0 (0.0)

## Supplementary Materials

The following are available online at https://www.mdpi.com/1660-4601/17/24/9178/s1, Figure S1: RAD7 Photo, Table S1: Indoor and Soil Gas Radon Levels Example Report Back, Booklet S1: Radon Test Kit Booklet.
