# Supplementary material for: High School Students as Citizen Scientists to Decrease Radon Exposure
Source: Int J Environ Res Public Health. 2020 Dec 8;17(24):9178. doi: 10.3390/ijerph17249178 (PMC7763282; doi:10.3390/ijerph17249178)
Supplement: Supplementary file 1 [file ijerph-17-09178-s001.zip › SupplementaryFiles/Radon test kit booklet/Radon test kit booklet-Fairfield.pdf]

# RADON TEST KIT
## BOOKLET

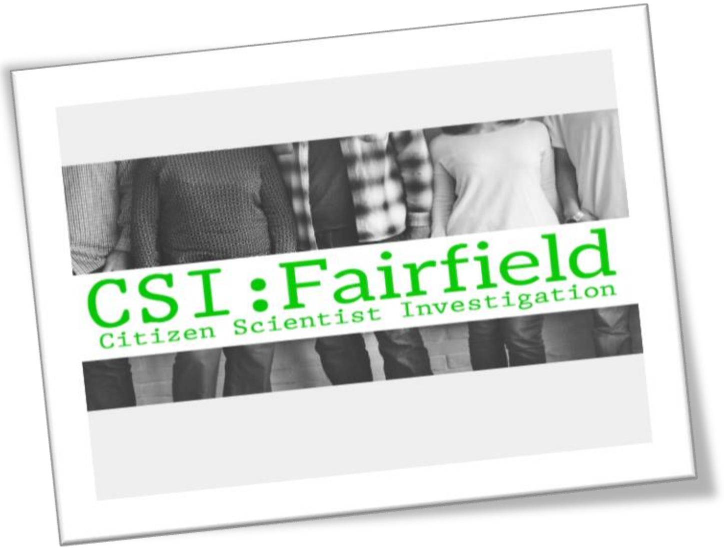


**Developed by:**
University of Kentucky’s NIEHS Environmental Health Center

National Institute of Environmental Health Sciences
University of Kentucky | Center for Appalachian Research in Environmental Sciences
University of Cincinnati
CEG Center for Environmental Genetics
Community Engagement Core

# 1 LEARN

## About Radon

### What is radon?

Radon is a naturally-occurring, radioactive gas that you can't see, smell or taste. It is hard to detect because there are no immediate health symptoms related to exposure. Testing is needed to know if radon is present in a home or building. If radon is found, radon levels can be fixed to lower health risks.

### Where does radon come from?

Radon comes from the breakdown of uranium in rock and soil. While it comes from natural sources, radon trapped inside buildings is not natural and can reach levels much higher than outdoor levels. Radon can enter any type of building, including homes where you and your family spend most of your time.

### How can radon affect your health?

Breathing air containing radon can cause lung cancer. Radon is the second leading cause of lung cancer in the United States, causing around 21,000 lung cancer deaths per year. While breathing radon is dangerous for everyone, it is more harmful when you also breathe tobacco smoke.

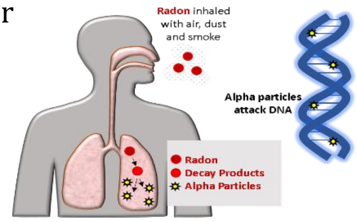


### How do I test my home?

Use the radon test kit instructions in this toolkit, following steps 2 through 5 to test and evaluate your home radon levels. **Do NOT open the plastic around the test kit until you are ready to begin testing.** Read all instructions before testing your home.

# 2 PREPARE
## Before Testing

### What does my radon toolkit include?

1. Radon test kit booklet
2. Radon test kit
3. Radon test information sheet
4. "Keep this page" sheet
5. Pre-paid postage envelope
6. Homeowner letter about soil sample collection
7. Radon and secondhand smoke fact sheet
8. County radon infographic sheet

**IMPORTANT: Keep all materials in a safe place.**

### Before testing:

1. Take the online survey at https://tinyurl.com/Radon-Fairfield.
2. Begin to fill out the "Radon test information sheet."
3. Keep all exterior doors and windows closed at least 12 hours before starting and during the test (normal entry and exit of the home is permitted).

### Where to place your test kit:

- Lowest level of your home where people spend at least 7 hours per week
- 2-7 feet above the floor (e.g., on a table or bookshelf)
- At least 3 feet away from exterior doors and windows
- At least 6 inches from any wall or object taller than 6 inches
- Out of reach of children and pets

### Do NOT place your test kit:

- In an area with high humidity (ex: kitchen, bathroom)
- Near heating or air conditioning vents
- In direct heat or sunlight or where it will get wet

# 3 TEST
## Your Home

## Complete these Steps:

1. **Open the plastic bag** containing the radon test kit (this is how you start your test).
2. **Record the start date and time** on the “Radon test information sheet” included in your toolkit or register this information online at: www.DrHomeAir.com/Register.
3. Also, record the start date and time and your test kit serial number (on the charcoal side of your test kit) on the “Keep this page” for your records.
4. Place the test kit in the selected location (go back to page 3) with the **paper side up.**

## IMPORTANT:

1. **Do NOT puncture, tear, or rip the radon test kit.**
2. **Keep test kit in place for at least 2 full days (48 hours) and no longer than 4 full days (96 hours).**

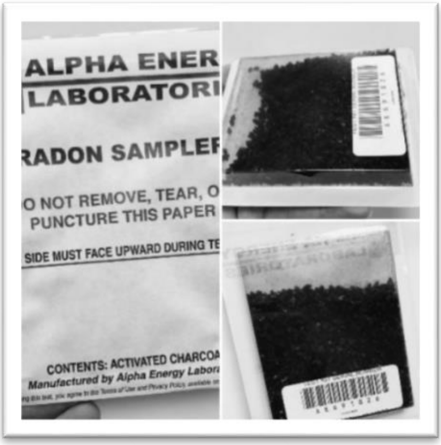

# 4 RETURN
## Test Kit to Lab

## Complete these steps to return your kit:

1. **Stop the test** after at least 2 full days (48 hours) and no longer than 4 full days (96 hours).

5. **Record the stop date and time** on the "Radon test information sheet" or online at www.DrHomeAir.com/Entry (if you started recording online) and **complete any missing information**. Also, record the stop date and time on your "Keep this page."

    **IMPORTANT: Stop date and time are required for the lab to analyze the results.**

2. Place completed "Radon test information sheet" and radon test kit inside the Pre-paid postage envelope and **seal the envelope** (this is how you stop your test).

3. **Mail the package immediately** to Alpha Energy Labs after sealing the envelope:

    2501 Mayes Road Suite #100
    Carrollton, TX 75006

*Contact **Nick Conley**, UK College of Nursing BREATHE, at **(859)257-7028 or nicholas.conley@uky.edu** if you have any questions.*

# 5 | EVALUATE
## The Collected Data

### How do I evaluate my home radon data?

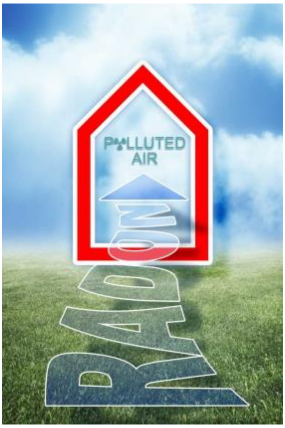


Radon is measured in picocuries per liter of air, or pCi/L. The Environmental Protection Agency (EPA) recommends fixing your home **if your radon value is 4 pCi/L or higher**. If your home tests at or above 4 pCi/L, you may want to take a follow-up test to be sure.

If you take part in the soil testing for radon, we will have a conversation with you about how it compares with the radon level in your home.

### What do I do if my home has high radon?

- ✓ **Talk** with a health care provider about your results.
- ✓ **Fix** your home if the level is high. Call a certified radon professional.
- ✓ **Ask** people to smoke outside and away from entryways.
- ✓ **Tell** your loved ones about the dangers of radon and tobacco smoke.

### How do I fix the radon level in my home?

Call a certified radon professional to install a radon mitigation system, which will pull radon from underneath your home and vent it outside. A certified radon mitigation professional will customize the right system for your home, depending on the house foundation, structure and design.

# 6 RESOURCES

## Sample Radon Test Information Sheet

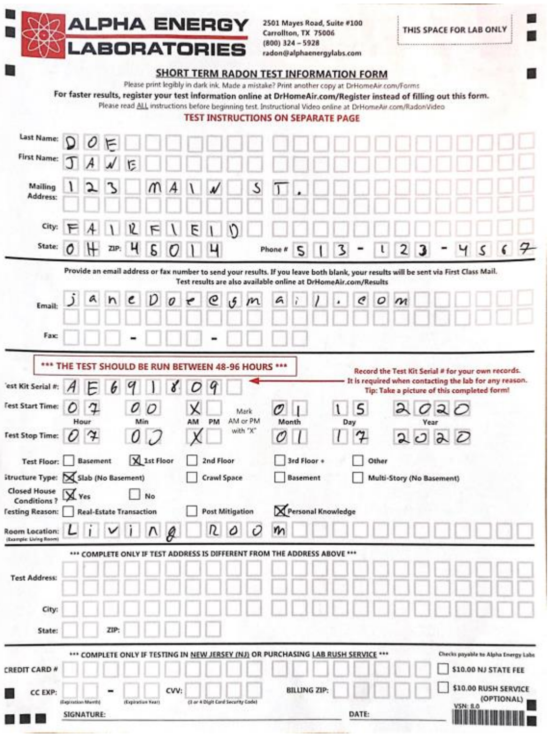

ALPHA ENERGY LABORATORIES
2501 Mayes Road, Suite #100
Carrollton, TX 75006
(800) 324 – 5928
radon@alphaenergylabs.com

THIS SPACE FOR LAB ONLY

SHORT TERM RADON TEST INFORMATION FORM

Please print legibly in dark ink. Made a mistake? Print another copy at DrHomeAir.com/Forms
For faster results, register your test information online at DrHomeAir.com/Register instead of filling out this form.
Please read ALL instructions before beginning test. Instructional Video online at DrHomeAir.com/RadonVideo
TEST INSTRUCTIONS ON SEPARATE PAGE

Last Name: DOE
First Name: JANE
Mailing Address: 123 MAIN ST.
City: FAIRFIELD
State: OH ZIP: 45014 Phone # 513 - 123 - 4567

Provide an email address or fax number to send your results. If you leave both blank, your results will be sent via First Class Mail.
Test results are also available online at DrHomeAir.com/Results

Email: janeDoe@gmail.com
Fax: - -

*** THE TEST SHOULD BE RUN BETWEEN 48-96 HOURS ***

Test Kit Serial #: AE691809

Record the Test Kit Serial # for your own records.
It is required when contacting the lab for any reason.
Tip: Take a picture of this completed form!

Test Start Time: 07 (Hour) 00 (Min) X AM PM (Mark AM or PM with "X") 01 (Month) 15 (Day) 2020 (Year)
Test Stop Time: 07 00 X AM PM 01 17 2020

Test Floor: ☐ Basement ☒ 1st Floor ☐ 2nd Floor ☐ 3rd Floor + ☐ Other
Structure Type: ☒ Slab (No Basement) ☐ Crawl Space ☐ Basement ☐ Multi-Story (No Basement)
Closed House Conditions ? ☒ Yes ☐ No
Testing Reason: ☐ Real-Estate Transaction ☐ Post Mitigation ☒ Personal Knowledge
Room Location (Example: Living Room): Living Room

*** COMPLETE ONLY IF TEST ADDRESS IS DIFFERENT FROM THE ADDRESS ABOVE ***

Test Address:
City:
State: ZIP:

*** COMPLETE ONLY IF TESTING IN NEW JERSEY (NJ) OR PURCHASING LAB RUSH SERVICE ***

Checks payable to Alpha Energy Labs

CREDIT CARD #
☐ $10.00 NJ STATE FEE
CC EXP: (Expiration Month) - (Expiration Year) CVV: (3 or 4 Digit Card Security Code) BILLING ZIP:
☐ $10.00 RUSH SERVICE (OPTIONAL)
SIGNATURE: DATE:
VSN: 8.0

## For Additional Information, visit:

- University of Kentucky BREATHE website at http://www.uky.edu/breathe/
- EPA's Citizen's Guide to Radon at https://www.epa.gov/radon/citizens-guide-radon-guide-protecting-yourself-and-your-family-radon
- OHIO EPA Radon Webpage at https://odh.ohio.gov/wps/portal/gov/odh/know-our-programs/radon-education-and-licensing-program/about-radon/

# 7 CHECKLIST

## Check when Complete!

## 1: LEARN about radon

- ☐ Read about radon and all instructions before testing.

## 2: PREPARE before testing

- ☐ **Take the survey** at https://tinyurl.com/Radon-Fairfield.
- ☐ Begin filling out the "Radon test information sheet."
- ☐ Keep all exterior doors and windows closed at least 12 hours before starting and during the test.
- ☐ Choose the best place to put your radon test.

## 3: TEST your home

- ☐ **Open the plastic bag** containing the radon test kit.
- ☐ Record the start date and time on the "Radon test information sheet."
- ☐ Place the test kit with the **paper side up**.

## 4: RETURN test kit to lab

- ☐ **Stop the test** after at least 2 full days.
- ☐ **Record the stop date and time** on the "Radon test information sheet."
- ☐ Place completed "Radon test information sheet" and test kit inside the Pre-paid postage envelope and **seal it**.
- ☐ **Mail the package immediately** to Alpha Energy Labs.

## 5: EVALUATE the collected data

- ☐ Review data from home and/or soil measurements and identify next steps to lower radon risk.
