# Supplementary figures and images for: High School Students as Citizen Scientists to Decrease Radon Exposure
Source: Int J Environ Res Public Health. 2020 Dec 8;17(24):9178. doi: 10.3390/ijerph17249178 (PMC7763282; doi:10.3390/ijerph17249178)

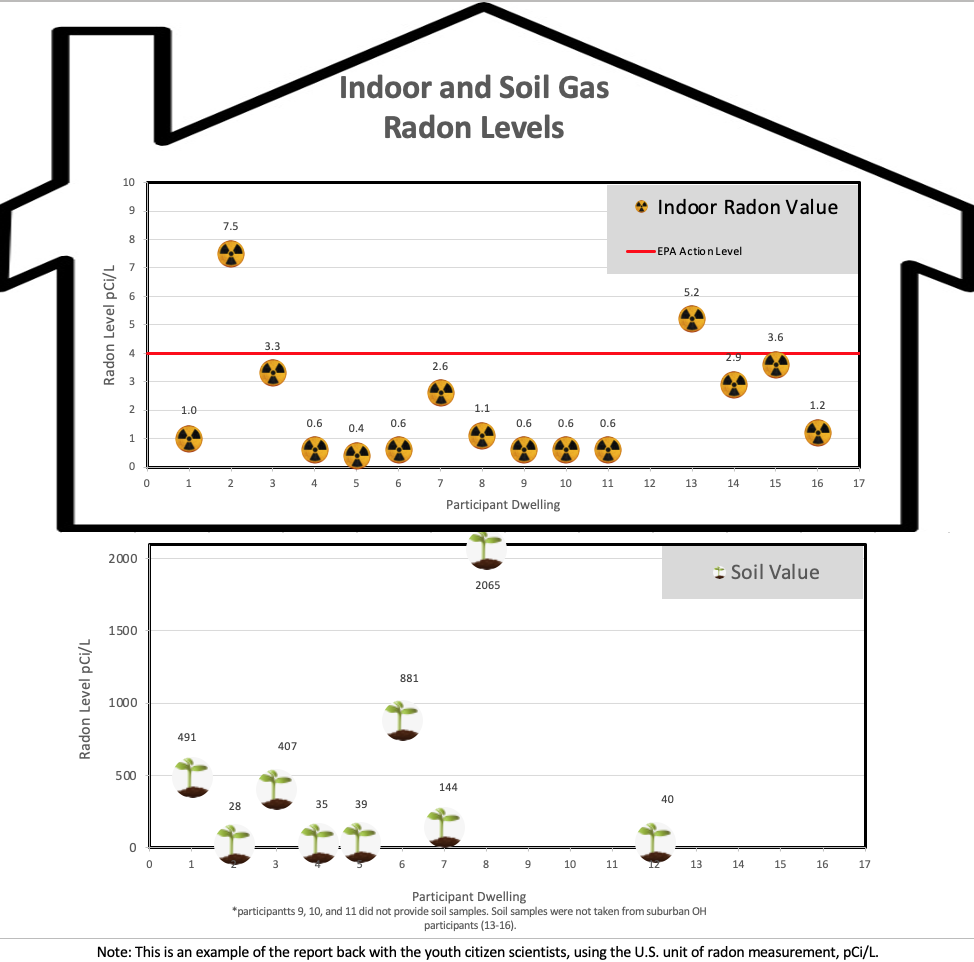

Supplement: Supplementary file 1 [file ijerph-17-09178-s001.zip › SupplementaryFiles/Example of report back/RadonDataFigure Community Report Back.png]

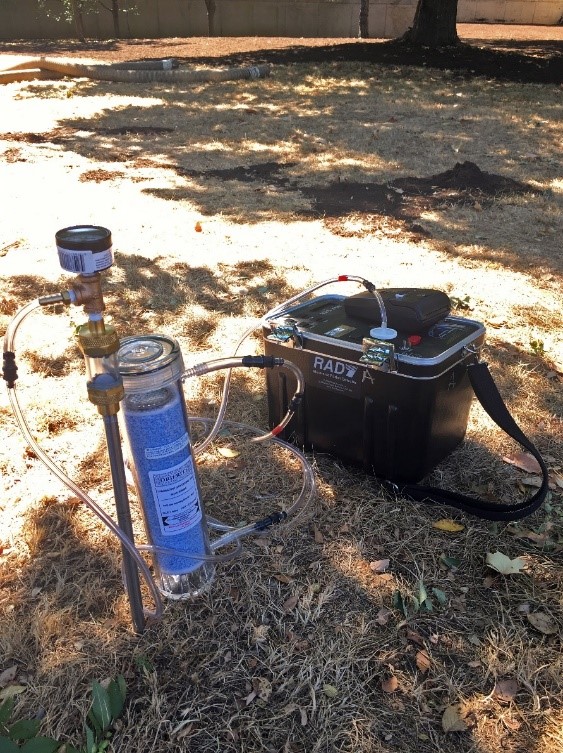

Supplement: Supplementary file 1 [file ijerph-17-09178-s001.zip › SupplementaryFiles/Figure RAD7 Photo/Figure RAD7 Photo.jpg]
